# Supplementary material for: Iterative improvement in the automatic modular design of robot swarms
Source: PeerJ Comput Sci. 2020 Dec 7;6:e322. doi: 10.7717/peerj-cs.322 (PMC7924708; doi:10.7717/peerj-cs.322)
Supplement: Supplemental Information 3 [file peerj-cs-06-322-s003.zip › argos3/doc/api/standalone/a00450_source.html]

ARGoS: plugins/robots/foot-bot/control\_interface/ci\_footbot\_turret\_actuator.cpp Source File


- Main Page
- Related Pages
- Namespaces
- Classes
- Files

- File List
- File Members

# plugins/robots/foot-bot/control\_interface/ci\_footbot\_turret\_actuator.cpp

Go to the documentation of this file.

```
00001 
00007 #include "ci_footbot_turret_actuator.h"
00008 
00009 #ifdef ARGOS_WITH_LUA
00010 #include <argos3/core/wrappers/lua/lua_utility.h>
00011 #endif
00012 
00013 namespace argos {
00014 
00015   /****************************************/
00016   /****************************************/
00017 
00018   const CRange<SInt32> CCI_FootBotTurretActuator::SPEED_RANGE(-4,4);
00019   const CRange<Real>   CCI_FootBotTurretActuator::NORMALIZED_SPEED_RANGE(-1.0,1.0);
00020 
00021   /****************************************/
00022   /****************************************/
00023 
00024 #ifdef ARGOS_WITH_LUA
00025   int LuaTurretSetRotation(lua_State* pt_lua_state) {
00026     /* Check parameters */
00027     if(lua_gettop(pt_lua_state) != 1) {
00028       return luaL_error(pt_lua_state, "robot.turret.set_rotation() expects 1 argument");
00029     }
00030     luaL_checktype(pt_lua_state, 1, LUA_TNUMBER);
00031     /* Perform action */
00032     CLuaUtility::GetDeviceInstance<CCI_FootBotTurretActuator>(pt_lua_state, "turret")->
00033       SetRotation(CRadians(lua_tonumber(pt_lua_state, 1)));
00034     return 0;
00035   }
00036 #endif
00037 
00038 #ifdef ARGOS_WITH_LUA
00039   int LuaTurretSetRotationSpeed(lua_State* pt_lua_state) {
00040     /* Check parameters */
00041     if(lua_gettop(pt_lua_state) != 1) {
00042       return luaL_error(pt_lua_state, "robot.turret.set_rotation_speed() expects 1 argument");
00043     }
00044     luaL_checktype(pt_lua_state, 1, LUA_TNUMBER);
00045     /* Perform action */
00046     CLuaUtility::GetDeviceInstance<CCI_FootBotTurretActuator>(pt_lua_state, "turret")->
00047       SetRotationSpeed(lua_tonumber(pt_lua_state, 1));
00048     return 0;
00049   }
00050 #endif
00051 
00052 #ifdef ARGOS_WITH_LUA
00053   int LuaTurretSetSpeedControlMode(lua_State* pt_lua_state) {
00054     /* Check parameters */
00055     if(lua_gettop(pt_lua_state) != 0) {
00056       return luaL_error(pt_lua_state, "robot.turret.set_speed_control_mode() expects no arguments");
00057     }
00058     /* Perform action */
00059     CLuaUtility::GetDeviceInstance<CCI_FootBotTurretActuator>(pt_lua_state, "turret")->
00060       SetSpeedControlMode();
00061     return 0;
00062   }
00063 #endif
00064 
00065 #ifdef ARGOS_WITH_LUA
00066   int LuaTurretSetPositionControlMode(lua_State* pt_lua_state) {
00067     /* Check parameters */
00068     if(lua_gettop(pt_lua_state) != 0) {
00069       return luaL_error(pt_lua_state, "robot.turret.set_position_control_mode() expects no arguments");
00070     }
00071     /* Perform action */
00072     CLuaUtility::GetDeviceInstance<CCI_FootBotTurretActuator>(pt_lua_state, "turret")->
00073       SetPositionControlMode();
00074     return 0;
00075   }
00076 #endif
00077 
00078 #ifdef ARGOS_WITH_LUA
00079   int LuaTurretSetPassiveMode(lua_State* pt_lua_state) {
00080     /* Check parameters */
00081     if(lua_gettop(pt_lua_state) != 0) {
00082       return luaL_error(pt_lua_state, "robot.turret.set_passive_mode() expects no arguments");
00083     }
00084     /* Perform action */
00085     CLuaUtility::GetDeviceInstance<CCI_FootBotTurretActuator>(pt_lua_state, "turret")->
00086       SetPassiveMode();
00087     return 0;
00088   }
00089 #endif
00090 
00091   /****************************************/
00092   /****************************************/
00093 
00094   void CCI_FootBotTurretActuator::SetActiveWithRotation(const CRadians& c_angle) {
00095     SetPositionControlMode();
00096     SetRotation(c_angle);
00097   }
00098 
00099   /****************************************/
00100   /****************************************/
00101 
00102   void CCI_FootBotTurretActuator::SetSpeedControlMode() {
00103     SetMode(MODE_SPEED_CONTROL);
00104   }
00105 
00106   /****************************************/
00107   /****************************************/
00108 
00109   void CCI_FootBotTurretActuator::SetPositionControlMode() {
00110     SetMode(MODE_POSITION_CONTROL);
00111   }
00112 
00113   /****************************************/
00114   /****************************************/
00115 
00116   void CCI_FootBotTurretActuator::SetPassiveMode() {
00117     SetMode(MODE_PASSIVE);
00118   }
00119 
00120   /****************************************/
00121   /****************************************/
00122 
00123 #ifdef ARGOS_WITH_LUA
00124   void CCI_FootBotTurretActuator::CreateLuaState(lua_State* pt_lua_state) {
00125     CLuaUtility::OpenRobotStateTable(pt_lua_state, "turret");
00126     CLuaUtility::AddToTable(pt_lua_state, "_instance", this);
00127     CLuaUtility::AddToTable(pt_lua_state, "set_rotation", &LuaTurretSetRotation);
00128     CLuaUtility::AddToTable(pt_lua_state, "set_rotation_speed", &LuaTurretSetRotationSpeed);
00129     CLuaUtility::AddToTable(pt_lua_state, "set_position_control_mode", &LuaTurretSetPositionControlMode);
00130     CLuaUtility::AddToTable(pt_lua_state, "set_speed_control_mode", &LuaTurretSetSpeedControlMode);
00131     CLuaUtility::AddToTable(pt_lua_state, "set_passive_mode", &LuaTurretSetPassiveMode);
00132     CLuaUtility::CloseRobotStateTable(pt_lua_state);
00133   }
00134 #endif
00135 
00136   /****************************************/
00137   /****************************************/
00138 
00139 }
```

---

Generated on 10 Jul 2018 for ARGoS by 
 1.6.1
